# The Role of Enoyl Reductase in the Monacolin K Biosynthesis Pathway in *Monascus* spp.

**DOI:** 10.3390/jof11030199

**Published:** 2025-03-04

**Authors:** Tingting Yao, Xiaodi Wang, Fusheng Chen

**Affiliations:** National Key Laboratory of Agricultural Microbiology, Hubei International Scientific and Technological Cooperation Base of Traditional Fermented Foods, College of Food Science and Technology, Huazhong Agricultural University, Wuhan 430070, China; ytt1116tty@163.com (T.Y.); wang-xiaodi@webmail.hzau.edu.cn (X.W.)

**Keywords:** *Monascus pilosus*, monacolin K, gene cluster, biosynthetic pathway

## Abstract

Monacolin K (MK), a secondary metabolite produced by *Monascus* spp. with the ability to inhibit cholesterol production, is structurally identical to lovastatin produced by *Aspergillus terreus.* In the lovastatin biosynthetic pathway, the polyketide synthase (PKS) encoded by *lovB* must work together with the enoyl reductase encoded by *lovC* to ensure lovastatin production. However, it is unclear whether *mokA* and *mokE* in the MK gene cluster of *Monascus* spp., both of which are highly homologous to *lovB* and *lovC*, respectively, also have the same functions for MK biosynthesis. In the current study, the high-yielding MK *M. pilosus* MS-1 was used as the research object, and it was found that the enoyl reductase domain of MokA may be non-functional due to the lack of amino acids at active sites, a function that may be compensated for by MokE in the MK synthesis pathway. Then, the *mokE*-deleted (Δ*mokE*), -complemented (Δ*mokE::mokE*), and -overexpressed (*PgpdA-mokE*) strains were constructed, and the results showed that Δ*mokE* did not produce MK, and Δ*mokE::mokE* restored MK synthesis, while the ability of *PgpdA-mokE* to produce MK was increased by 32.1% compared with the original strain MS-1. These results suggest that the MokA synthesized by *Monascus* spp. must be assisted by MokE to produce MK, just as lovastatin produced by *A. terreus*, which provides clues for further genetic engineering to improve the yield of MK in *Monascus* spp.

## 1. Introduction

*Monascus* spp., used in China and other Asian countries for nearly 2000 years, are medicinal and edible dual-use fungi [1,2]. They can produce many secondary metabolites such as monacolin K (MK), which has lipid-lowering properties [3,4], *Monascus* pigment, a natural food colorant [5,6] (Figure S1), and a toxic secondary metabolite, citrinin [7,8]. Among them, MK, also known as lovastatin, a cholesterol synthesis inhibitor, was isolated from the fermentation products of *M. ruber* and *A. terreus* in the late 1970s [9,10]. In addition to inhibiting cholesterol synthesis, MK also has a great inhibitory effect on cancer cells, such as those of colon cancer, prostate cancer, breast cancer, and acute myeloid leukemia [11,12,13,14], so products high in MK content from *Monascus* spp. have been developed into drugs and functional foods that regulate blood lipids [1,15,16].

However, the ability of *Monascus* spp. to synthesize MK is quite limited [17], resulting in a long fermentation time and low efficiency in yielding MK-related products, so researchers have tried to improve the ability of *Monascus* spp. to produce MK through strain screening, mutagenesis breeding, optimization of media composition, and fermentation conditions, but the results are not ideal [4,18,19].

**Figure 1:**
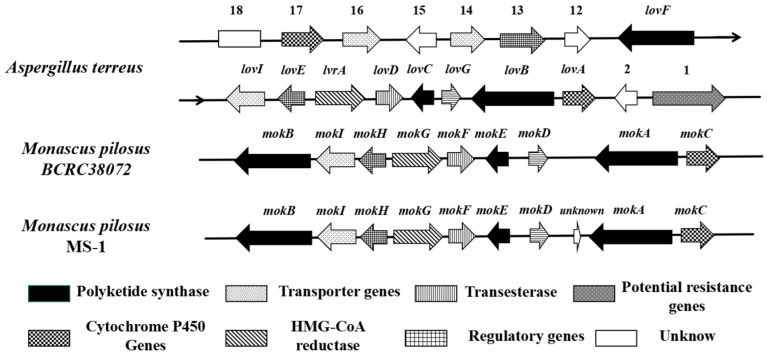
Comparison of monacolin K gene clusters in *A. terreus* and *M. pilosus*.

**Figure 2:**
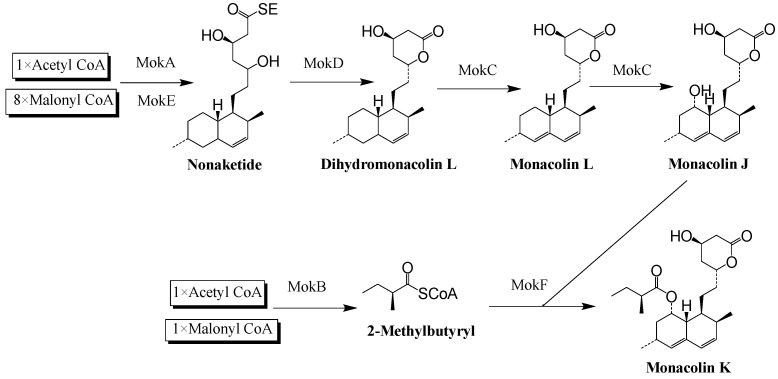
The biosynthetic pathway of monacolin K. MokA: polyketide synthase; MokB: polyketide synthase; MokC: P450 monooxygenase; MokD: oxidoreductase; MokE: enoyl reductase; MokF: transesterase.

At the beginning of this century, a nine-gene (*mokA*-*mokI*) MK gene cluster was found in the genome of *M. pilosus* BCRC38072 (GenBank: DQ176595.1), in which the MK-related genes are highly homologous to the corresponding genes in the lovastatin gene cluster of *A. terreus* (GenBank: AH007774.2, AF151722; Figure 1 and Figure 2 and Table S1) [20,21,22]. This provides the possibility of improving the ability of *Monascus* spp. to synthesize MK by modifying the biosynthetic pathway of MK. In recent years, Zhang et al. [23,24,25] have used *M. purpureus* as an experimental strain to construct *mokC*-, *mokD*-, *mokE*-, *mokF*-, *mokH*-, and *mokI*-overexpressed strains, and their yields of MK were increased by 234.3%, 220.8%, 89.5%, 74.19%, 82%, and 10%, respectively, compared with the original strain. Overexpression of the *mokH* and *mokI* genes in the MK gene cluster of *M. pilosus* can also increase MK yield [26,27].

In the biosynthesis pathway of lovastatin of *A. terreus*, LovB (lovastatin nonaketide synthase; MokA homologous enzyme) with multiple domains is a key synthase of lovastatin, in which the function of the enoyl reductase domain is compensated for by LovC (enoyl reductase; MokE homologous enzyme) due to the lack of amino acid active sites and conserved motif, thus ensuring the synthesis of lovastatin [28,29,30,31]. Is the enoyl reductase domain of MokA active? If it is not active, can MokA function be compensated for by the enoyl reductase MokE in the MK synthesis pathway? None of these issues is currently clear, and no study has been reported yet.

Therefore, in the current study, *M. pilosus* MS-1 with high MK production and without citrinin production was taken as the research object [4], and it was found that the enoyl reductase domain of MokA was also inactive, and its function was compensated for by MokE in the MK synthesis pathway, through deleting, complementing, and overexpressing *mokE*, meaning that MokE can indeed compensate for the enoyl reductase domain function of MokA to ensure MK biosynthesis in *M. pilosus* MS-1. Our research results provide a clue to enhancing the yield of MK in *Monascus* spp.

## 2. Materials and Methods

### 2.1. Strains, Plasmids, and Media

*M. pilosus* MS-1 (CCTCCM 2013295), which can produce high levels of MK without citrinin, is deposited in the culture bank of the State Key Laboratory of Agricultural Microbiology in Wuhan, China [4]. All strains used in this study are listed in Table 1, and plasmids pSKH, pKNI, p21, and pCAMBIA3300 for the construction of recombinant vectors are preserved in our laboratory. Luria–Bertani (LB) medium, induction medium (IM), and co-culture induction medium (Co-IM) were used for the transformation of strains, while potato dextrose agar (PDA) medium was utilized for the analysis of MP production [17], and a seed medium and a soybean–rice flour medium were used for MK production [18]. Hygromycin B and G418 (Sigma-Aldrich, Shanghai, China) were used in the selection of transformants [32].

### 2.2. Domain Analysis of MokA and MokE from M. pilosus MS-1

**Figure 3:**
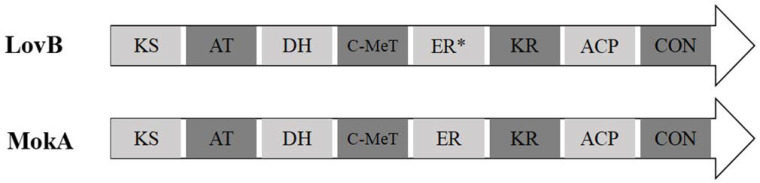
Domains of MokA from *M. pilosus* MS-1 and LovB of *A. terreus.* KS: ketoacyl synthase; AT: acyltransferase; DH: dehydratase; C-MeT: carbon methyl transferase; ER: enoyl reductase (ER* means inactive); KR: ketoreductase; ACP: acyl carrier protein; CON: condensation (participate in the Diels–Alder reaction during the synthesis of nonaketide from MokA and MokE).

**Figure 4:**
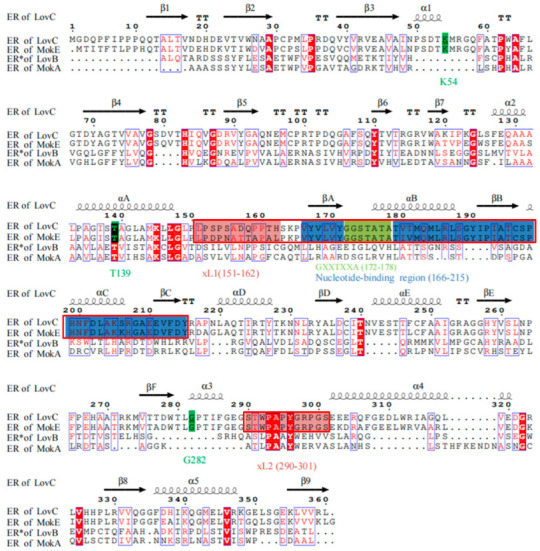
Alignments of ER domains of MokA and LovB to those of MokE and LovC. α: α helix; β: β fold; xL1 (151–162) and xL2 (290–301): the extension loop; nucleotide-binding region (166–215): nicotinamide adenine dinucleotide phosphate (NADP)-binding region; GXXTXXA (172–178): the conserved motif. The color of the background is intended to correspond to the color of the comment.

**Figure 5:**
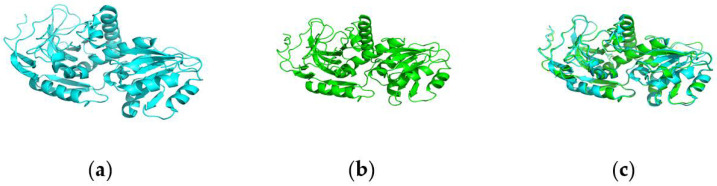
Three-dimensional structure diagrams of LovC and MokE. (**a**) Three-dimensional structure diagram of LovC; (**b**) three-dimensional structure diagram of MokE; (**c**) comparison of the overlay of the 3D structure of LovC and MokE.

The amino acid sequences of MokA (Q3S2T9) and MokE (Q3S2U1) in the MK gene cluster of *M. pilosus* MS-1 were downloaded from the NCBI database (https://www.ncbi.nlm.nih.gov/, accessed on 6 December 2024) [21], and the conserved domain database CDD (https://www.ncbi.nlm.nih.gov/Structure/cdd/wrpsb.cgi, accessed on 6 December 2024) was applied to predict the domains (Figure 3), and the differences between the conserved domains of MokE and the enoyl reductase domains of MokA were analyzed and compared (Figure 4 and Figure 5).

### 2.3. Deletion, Complementation, and Overexpression of the mokE Gene in M. pilosus MS-1

**Figure 6:**
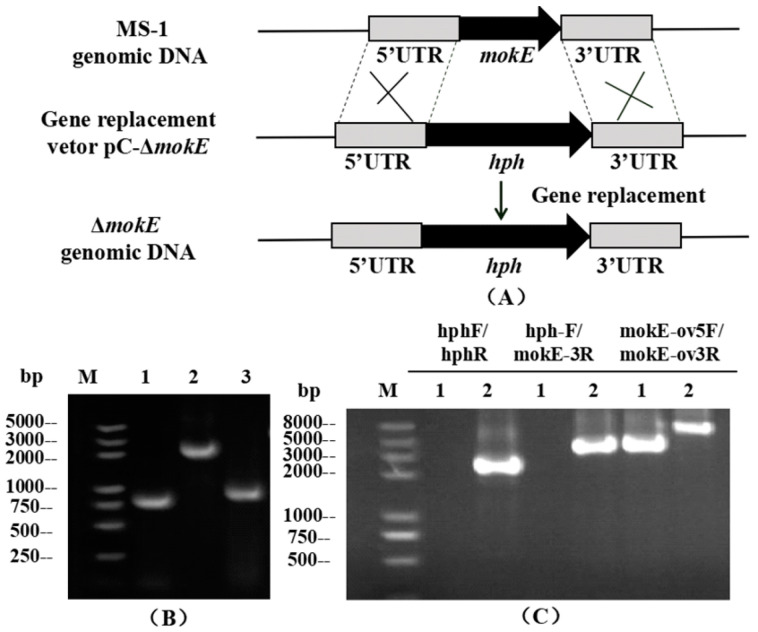
Schematic diagram of the *mokE* deletion strain and PCR validation diagram. (**A**) Schematic diagram of the homologous recombination strategy of the *mokE* deletion strain. (**B**) PCR validation of *mokE* deletion vector constructed by seamless cloning. Lane 1: 5’ flanking region of *mokE*, Lane 2: *hph* gene fragment, Lane 3: 3’ flanking region of *mokE*. (**C**) PCR validation image of *mokE* deletion strains. Using 3 pairs of primers, PCR amplification revealed different fragments in different strains. Lane 1: MS-1 strain; Lane 2: Δ*mokE* strain.

**Figure 7:**
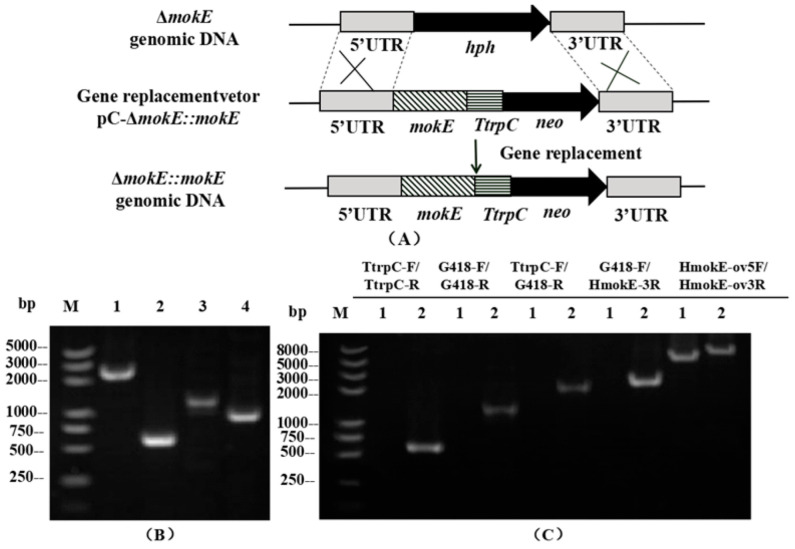
Schematic diagram of the *mokE* complementation strain and PCR validation diagram. (**A**) Schematic diagram of the homologous recombination strategy of an *mokE* complementation strain. (**B**) PCR validation of *mokE* complementation vector constructed by seamless cloning. Lane 1: 5’ flanking region of mokE (containing *mokE* open reading frame region), Lane 2: *TtrpC* fragment, Lane 3: *neo* gene fragment, Lane 4: 3’ flanking region of *mokE*. (**C**) PCR validation image of *mokE* complementation strains. Using 5 pairs of primers, PCR amplification revealed different bands in different strains. Lane 1: Δ*mokE* strain; Lane 2: Δ*mokE::mokE* strain.

**Figure 8:**
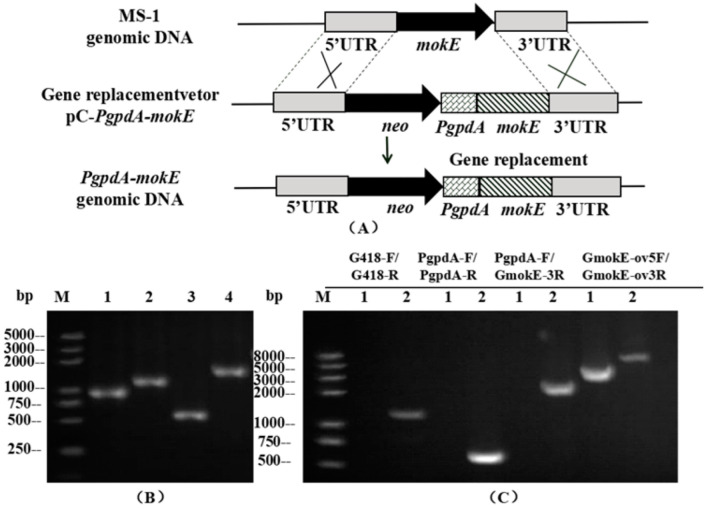
Schematic diagram of the *mokE* overexpression strain and PCR validation diagram. (**A**) Schematic diagram of the homologous recombination strategy of an *mokE* overexpression strain. (**B**) PCR validation of *mokE* overexpression vector constructed by seamless cloning. Lane 1: 5’ flanking region of *mokE*, Lane 2: *neo* gene fragment, Lane 3: *PgpdA* gene fragment, Lane 4: 3’ flanking region of *mokE* (containing *mokE* open reading frame region). (**C**) PCR validation image of *mokE* overexpression strains. Using 4 pairs of primers, PCR amplification revealed different bands in different strains. Lane 1: MS-1 strain; Lane 2: the putative *PgpdA-mokE* strain.

In order to analyze the function of *mokE*, deletion, complementation, and overexpression of *mokE* were performed according to the principle of homologous recombination, and their construction processes are shown in Figure 6A, Figure 7A and Figure 8A, respectively. The relative primers used are shown in Table S2.

Construction of the *mokE* gene deletion vector: 5’ flanking regions (814 bp, amplified with the primer pair mokE-5F and mokE-5R), hygromycin B resistance gene (*hph*, 2137 bp, amplified from plasmid pSKH with the primer pair hph-F and hph-R), 3’ flanking regions (913 bp, amplified with the primer pair mokE-3F and mokE-3R), and plasmid pCAMBIA3300 (digested with *Xba*I and *Hind*III) were obtained by seamless clonal splicing to obtain the deletion vector pC-Δ*mokE* [33].

Construction of the *mokE* gene complement vector: 5’ flanking regions (containing *mokE* open reading frame, 2282 bp, amplified with the primer pair HmokE-5F and HmokE-5R), terminator gene (*TtrpC*, 597 bp, amplified from plasmid pSKH with the primer pair TtrpC-F and TtrpC-R), neomycin phosphotransferase resistance gene (*neo*, 1221 bp, amplified from plasmid pKN1 with the primer pair G418-F and G418-R), 3’ flanking regions (913 bp amplified with the primer pair HmokE-3F and HmokE-3R), and plasmid pCAMBIA3300 (digested with *Xba*I. and *Hind*III) were obtained by seamless clonal splicing to obtain the compensation vector pC-Δ*mokE::mokE*.

Construction of *mokE* gene overexpression vector: 5’ flanking regions (910 bp, amplified with the primer pair GmokE-5F and GmokE-5R), neomycin phosphotransferase resistance gene (*neo*, 1221 bp, amplified from plasmid pKN1 with the primer pair G418-F and G418-R), *gpdA* promoter (604 bp, amplified from plasmid p21 with the primer pair gpdA-F and gpdA-R), 3’ flanking regions (containing *mokE* open reading frame, 1779 bp, amplified with the primer pair GmokE-3F and GmokE-3R), and the plasmid pCAMBIA3300 (digested with *Xba*I and *Hind*III) were obtained by seamless clonal splicing to obtain the overexpression vector pC-*PgpdA-mokE*.

The above-mentioned three vectors were transformed into *Agrobacterium tumefaciens* EHA105 by a freeze–thaw method [34].

The *A. tumefaciens* EHA105 strain containing pC-Δ*mokE* and pC-*PgpdA-mokE* was then inoculated into IM medium, incubated at 28 °C to achieve an OD600 nm value of 0.8–1.2 for *A. tumefaciens*, and coincubated with MS-1 in IM for 6 h (28 °C), then the mixed strains were coated in Co-IM medium for transformation, and finally the putative deletion strain (Δ*mokE*) and overexpression strains (*PgpdA-mokE*) were verified by PCR. The recombinant vector pC-Δ*mokE::mokE* in *A. tumefaciens* EHA105 was co-cultured with the Δ*mokE* strain to obtain the putative complementation strain (Δ*mokE::mokE*) [32,35].

### 2.4. Real-Time qPCR Analysis

MS-1, Δ*mokE*, Δ*mokE::mokE*, and *PgpdA-mokE* strains were cultured on PDA media for 11 days at 28 °C, and their spores were collected, and the spore concentrations were adjusted to 10^6^ cfu/mL. Then, 100 μL of the spore solutions was spread on PDA media coated with cellophane and incubated at 28 °C, and the mycelia were collected at days 3, 6, and 9 for RNA extraction, and the RNA was converted to cDNA. The *beta-actin* gene was used as the internal reference gene to perform RT-qPCR of MK- and MP-related genes in MS-1 [32]. The relevant primers are listed in Table S3.

### 2.5. Analysis of Monacilin K and Monascus Pigments

#### 2.5.1. Monacolin K Analysis

The spore solutions (10^6^ cfu/mL) of MS-1, Δ*mokE*, Δ*mokE::mokE,* and *PgpdA-mokE* strains were inoculated into the seed media at 10% (*v*/*v*) rate and incubated at 30 °C, 110 rpm for 36 h. After that, the broths were transformed into the solid medium of rice–soybean flour at 13% (*v*/*w*) rate, incubated at 30 °C for 60 h, and then transferred to 24 °C to cultivate for 14 days. Samples were taken on the 7th, 10th, and 14th days, dried at 45 °C to the constant weight, and ground into 100 mesh powder [18].

The 20 mg powder sample was immersed in 1.5 mL of 75% (*v*/*v*) ethanol and sonicated (KQ-250B, Kunshan, China) for 1 h, and centrifuged at 12,000 rpm for 10 min; then, the supernatant was filtered using a 0.22 μm microporous membrane for running HPLC (LC-20A, DaoJin, Japan), under the following conditions: acetonitrile and 0.5% phosphoric acid (60:40) used as mobile phases, flow rate 1.0 mL/min, column temperature 30 °C, and detection wavelength 238 nm (Inertsil ODS-3 4.6 × 250 mm column) [4,17]; Figure S2.

#### 2.5.2. MPs Analysis

A total of 200 μL of MS-1, Δ*mokE*, Δ*mokE::mokE*, and *PgpdA-mokE* spores (10^6^ cfu/mL) were evenly spread on PDA media coated with cellophane, and mycelia on the 3rd, 5th, 7th, 9th, and 11th days were collected for freeze-drying. After that, 20 mg of freeze-dried mycelia were sonicated in 1.5 mL of 80% methanol for 1 h, centrifuged at 12,000 rpm for 10 min, and then the supernatant was diluted with 80% methanol solution for the determination of absorbance values at 380, 470, and 520 nm [36].

## 3. Results

### 3.1. Comparison of Polyketide Synthases and Enoyl Reductases for the Biosynthesis of MK and Lovastatin from Monascus spp. and A. terreus, Respectively

In *A. terreus*, the enoyl reductase (ER) domain of polyketide synthase (LovB) loses its function due to the absence of amino acid active sites (Figure 1), and its function is compensated for by the enoyl reductase (LovC) in the lovastatin synthesis pathway [30]. In order to survey the similarity of MokA from *M. pilosus* MS-1 with LovB, their domains and amino acid sequences were compared, and the results are shown in Figure 3 and Table 2.

As can be seen from Figure 3 and Table 2, MokA and LovB all have eight domains: the KS, AT, DH, MeT, ER, KR, ACP, and CON domains, and the ER domains have the lowest similarity of 52.65%. It is known that the ER in LovB is inactive [37], but it is still unknown whether the ER domains in MokA are active, so the ER domains of MokA and LovB were compared to the conserved domains of their corresponding enoyl reductases MokE, and LovC, and the results are shown in Figure 4.

LovC (PDB ID: 3b6z; Figure 4) is the first-reported fungal trans-acting ER enzyme with a single crystal structure, consisting of two domains: a catalytic domain (residues 1–134, 283–363) and a cofactor-binding domain (Rossmann fold; residues 135–282), with two loops (xL1: 151–162 and xL2: 290–301) in the transition region between catalysis and cofactors, which are unique conserved features of trans-acting ER, and LovC is also an NADP-dependent ER consisting of βA, αB, βB, αC, and βC [37]. And its enoyl reduction is affected by the amino acid activities of K54, T139, and G282 [30].

As can be seen from Figure 4, the ER domains of MokE and LovC all have two extension loops (xL1 and xL2) and conserved nucleotide-binding regions (highly conserved motif: GXXTXXA), but all lack the active sites of catalytic substrates (K54, T139, G282), which mainly play a role in fixing the architecture [30,31,37]. Therefore, it is speculated that MokA from *M. pilosus* MS-1 may also need their MokE to synthesize MK, just as LovB requires LovC assistance to synthesize lovastatin [29,37].

In addition, the three-dimensional structural diagrams of MokE and LovC were analyzed with the help of AlphaFold3 and PyMOL [31,37], which showed that MokE were highly matched to LovC, implying that MokE and LovC were likely to have the same function and encode the same protein (Figure 5).

### 3.2. Construction of mokE-Deleted, -Complemented, and -Overexpressed Strains

In order to verify the role of MokE in the synthesis of MK from MS-1, the deletion, complementation, and overexpression strains of MokE were constructed based on the principle of homologous recombination (Figure 6, Figure 7 and Figure 8).

The recombinant plasmid pC-Δ*mokE* was transformed into MS-1 and then verified by PCR, and two putative deletion strains (Δ*mokE*) with hygromycin B resistance were obtained. As shown in Figure 6C, the 2.14 kb *hph* gene fragment and 3.05 kb fragment (containing the *hph* gene and the 3’ flanking region) can be amplified using the DNA genome of Δ*mokE* as a template, while none of these fragments were amplified using the DNA genome of MS-1 as a template. The fragment amplified by the primers MokE ov5F-MokE ov3R in MS-1 (3.09 kb) and Δ*mokE* (5.23 kb) was different.

The plasmid pC-Δ*mokE::mokE* was transformed into Δ*mokE* and then verified by PCR, and two complementation strains (Δ*mokE::mokE*) with G418 resistance were obtained. As shown in Figure 7C, the *TtrpC* fragment (0.6 kb), the *neo* gene fragment (1.21 kb), the 1.82 kb fragment (containing *TtrpC* and *neo* gene), and the 2.13 kb fragment (containing *neo* gene and 3’ flanking region) used the DNA genome of Δ*mokE::mokE* as a template, while no DNA band was amplified using the DNA genome of Δ*mokE* as a template. At the same time, the amplicon sizes of Δ*mokE* (5.49 kb) and Δ*mokE::mokE* (6.4 kb) differed when amplifying the extended homology arms (5’ flanking and 3’ flanking regions) using primer HMokE ov5F-HMokE ov3R.

The plasmid pC-*PgpdA-mokE* was transformed into MS-1 and then verified by PCR, and three overexpression strains (*PgpdA-mokE*) with G418 resistance were obtained. As shown in Figure 8C, the neo gene fragment (1.21 kb), the *PgpdA* gene fragment (0.6 kb), and the 2.08 kb fragment (containing the *PgpdA* gene, *mokE* open reading frame region, and 3’ flanking region) were amplified using the DNA genome of *PgpdA-mokE* as a template, while no DNA band was amplified using the DNA genome of MS-1 as a template.

At the same time, the amplicon sizes of MS-1 (3.31 kb) and *PgpdA-mokE* (5.13 kb) differed when amplifying the extended homology arms (5’ flanking and 3’ flanking regions) using primer GMokE ov5F-GMokE ov3R.

### 3.3. RT-qPCR Analyses of Key Genes in MK or MPs Gene Clusters of ΔmokE, ΔmokE::mokE, and PgpdA-mokE

**Figure 9:**
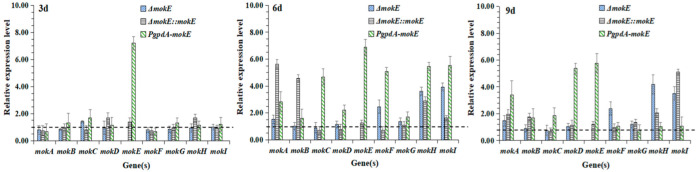
Transcriptional level analysis of key genes in MK gene clusters of Δ*mokE*, Δ*mokE::mokE,* and *PgpdA-mokE*. The expression level of the corresponding gene in MS-1 is 1, which is represented as y = 1.

RT-qPCR was used to analyze the transcription levels of *mokA*, *mokB*, *mokC*, *mokD*, *mokE*, *mokF*, *mokG*, *mokH*, and *mokI* genes in the MK gene clusters in MS-1, Δ*mokE*, Δ*mokE::mokE*, and *PgpdA-mokE*. As shown in Figure 9, the transcription level of the *mokE* gene in Δ*mokE* was undetectable, which further verified the successful deletion of the *mokE* gene. On the sixth and ninth days, the transcription levels of *mokH* and *mokI* were significantly higher than those of MS-1, and although the deletion of the *mokE* gene would lead to the inability to synthesize MK, it would not affect the synthesis of pyrone by *mokA* using acyl-CoA as a substrate [37], nor would it affect the synthesis of ethyl 2-methylbutyrate by *mokB* [22], so the deletion of the *mokE* gene was likely to increase the transcription levels of *mokH* and *mokI*.

The transcription levels of the *mokE* gene in the Δ*mokE::mokE* strain were 1.38, 1.27, and 1.23 times higher than those of MS-1 on the third, sixth, and ninth days, respectively. The transcription level of the *mokC* gene was always lower than that of MS-1. The transcription levels of *mokE* genes in *PgpdA-mokE* were 7.2, 6.88, and 5.76 times higher than those of MS-1 on the third, sixth, and ninth days, respectively. The transcription levels of the *mokA*, *mokB*, *mokC*, and *mokD* genes were higher than those of MS-1 on the sixth and ninth days, so the MK yield in *PgpdA-mokE* was higher than that of MS-1.

**Figure 10:**
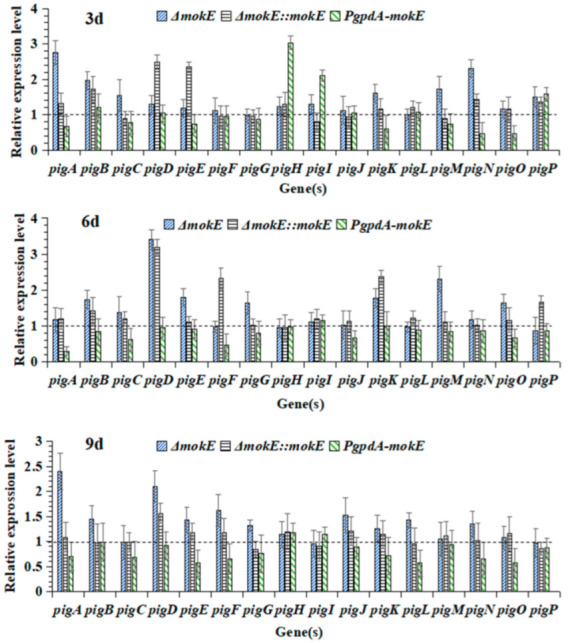
Transcriptional level analysis of key genes in MPs gene clusters of Δ*mokE*, Δ*mokE::mokE,* and *PgpdA-mokE*. The expression level of the corresponding gene in MS-1 is 1, which is represented as y = 1.

As can be seen from Figure 10, the expression levels of most genes in the MPs gene cluster of Δ*mokE* and Δ*mokE::mokE* were higher than those of MS-1 on the third, sixth, and ninth days, and the transcription level of the *pigD* gene was significantly higher than that of MS-1, which may also be the reason why the synthesis of *Monascus* pigment by Δ*mokE* and Δ*mokE::mokE* was higher than that of MS-1. The expression levels of most genes in the MPs gene cluster in *PgpdA-mokE* were lower than those of MS-1. In conclusion, the transcriptional results were consistent with the ability of MS-1 and its mutant strains to produce MPs on PDA media.

### 3.4. Analysis of MK and MPs of ΔmokE, ΔmokE::mokE, and PgpdA-mokE

**Figure 11:**
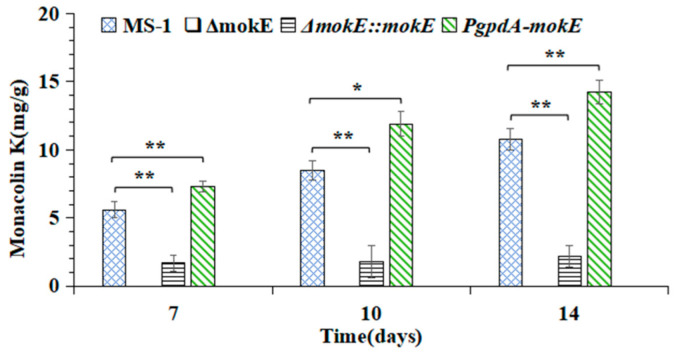
MK produced by *M. pilosus* MS-1, Δ*mokE*, Δ*mokE::mokE*, and *PgpdA-mokE.* Error bars represent the standard deviation between the three replicates. * means *p* < 0.05; ** means *p* < 0.01.

It is known that MS-1 can produce MK and MPs, but does not produce citrinin [4], and the determination of MK by fermentation in rice–soybean flour solid medium showed that the Δ*mokE* strain did not produce MK, and the Δ*mokE::mokE* strain could synthesize MK, but the yield of the Δ*mokE::mokE* strain was much lower than that of MS-1, only 1/5 of MS-1, and the yield of MK in the *PgpdA-mokE* strain increased by 32.1% on day 14, it was significantly (*p* < 0.01) higher than MS-1, as shown in Figure 11.

**Figure 12:**
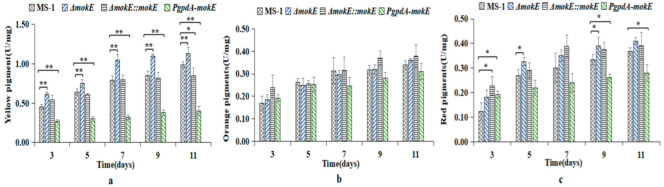
MPs produced by *M. pilosus* MS-1, Δ*mokE*, Δ*mokE::mokE*, and *PgpdA-mokE.* The yields of yellow MPs (**a**), orange MPs (**b**), and red MPs (**c**) of the strains were detected at 380, 470, and 520 nm. Error bars represent the standard deviation between the three replicates. * means *p* < 0.05; ** means *p* < 0.01.

The MPs produced by all strains will gradually accumulate with the increase in incubation time. Overall, there was no significant difference in orange pigment content among all strains. The production of yellow pigment in Δ*mokE* was significantly higher (*p* < 0.01) than that of MS-1, while the yellow pigment of *PgpdA-mokE* was extremely significant lower (*p* < 0.01) than that of MS-1, and the content of red pigment was also significant (*p* < 0.05) lower than that of MS-1 on the 3rd, 9th, and 11th days (Figure 12). Because the synthetic substrates of MK and MPs are the same, modifying the genes involved in the MK synthesis pathway can affect the production of MPs, resulting in a decrease in MK production and an increase in MP production [17].

## 4. Discussion

In the MK gene cluster of MS-1, the *mokE* gene encoding enoyl reductase is a unique presence, and when *mokE* was deleted, complemented, and overexpressed, MK could not be detected in the Δ*mokE* strain, while MK produced by *PgpdA-mokE* was higher than that of MS-1, and the Δ*mokE::mokE* strain could complement and synthesize MK (Figure 11). But the MK yield of the Δ*mokE::mokE* strain was much lower than that of MS-1, at only 1/5 of MS-1 (Figure 11). In order to explore the decrease in MK in the Δ*mokE::mokE* strain, the transcription levels of MK-related genes in this strain were determined, and the results revealed that the transcription levels of the *mokE* gene in the Δ*mokE::mokE* strain were 1.38, 1.27, and 1.23 times of those of MS-1 on the third, sixth, and ninth days, respectively, but the transcription levels of the *mokC* gene were lower than those of MS-1 (Figure 9). The decrease in MK production in the Δ*mokE::mokE* strain may be due to the decrease in *mokC* transcription level, as the *mokC* encoding P450 monooxygenase may affect the catalysis of dihydromonacrine L to monacolin J (Figure 2), and lead to a decrease in MK production [38]. In addition, during the process of supplementing *mokE* genes, genes related to MK biosynthesis may also be disrupted, resulting in a decrease in the MK production of Δ*mokE::mokE* [20].

In the current study, it is proved that polyketide synthase MokA of *M. pilosus* MS-1 contains KS, KR, MAT, DH, ER, MT, ACP, and CON, a total of eight domains, among which the ER domain is inactive due to the lack of active amino acid sites (Figure 4) [31], and its function is replaced by MokE. In order to investigate whether this phenomenon also exists in other *Monascus* spp., several strains of *M. pilosus* (BCRC38072, YDJ-1, YDJ2 and K104061) that can synthesize MK, were selected [21], and the domains of their MokAs were analyzed; it was found that none of the ER domains were active (Figures S3 and S4), and all of them contained the enoyl reductase MokE in the MK gene cluster. 

In addition to MokE and LovC, their homologous proteins are frequently found in other fungi (Figure S5), and they also play an important role in the synthesis of polyketide-nonribopeptides [38,39,40], such as tenellin [34,41], aspyridone A [42], and cytochalasin E/K [43].

In summary, MokE is essential in the MK biosynthesis of *M. pilosus* MS-1, as it can compensate for the function of the inactive ER domain in the MK polyketide synthase (MokA) to ensure its biosynthesis. Moreover, this phenomenon is also commonly observed in the biosynthesis of other polyketide compounds by other *Monascus* spp. and fungi, providing a novel clue for improving the biosynthesis of related polyketides by modifying MokE-like proteins from other fungi.

## Figures and Tables

**Table 1:** Strains used and constructed in this study.

Strains	Parents	Sources
MS-1	Wild type	Laboratory
Δ*mokE*	MS-1	This study
Δ*mokE::mokE*	Δ*mokE*	This study
*PgpdA-mokE*	MS-1	This study

**Table 2:** Homologies between MokA and LovB, and their domains.

MokA and Its Domains	LovB and Its Domains	Identities Between MokA and LovB (%)
MokA	LovB	76.88
KS	KS	89.02
AT	AT	77.64
DH	DH	78.71
MeT	MeT	94.00
ER	ER *	52.65
KR	KR	87.91
ACP	ACP	90.70
CON	CON	75.06

MokA: polyketide synthase; LovB: lovastatin nonaketide synthase. The annotations of KS, AT, DH, C-MeT, ER, KR, ACP, and CON are the same as Figure 3. * means inactive.

## Data Availability

The original contributions presented in this study are included in the article and Supplementary Materials. Further inquiries can be directed to the corresponding author.

## Supplementary Materials

The following supporting information can be downloaded at: https://www.mdpi.com/article/10.3390/jof11030199/s1, Figure S1. Alignments of ER domains of MokAs and LovB to those of MokEs and LovC; Figure S2. Domains of MokAs and MokEs from *M. pilosus* BCRC38072, MS-1, YDJ-1, YDJ-2, and K104061; Figure S3. ER in closely related fungal PKS. Figure S4. HPLC chromatograms of *M. pilosus MS-1*, *ΔmokE*, *ΔmokE::mokE*, and *PgpdA-mokE* in MK on day 7. Figure S5. The structure of six typical MP components. Table S1. Functional prediction of the MK biosynthesis gene and homology comparison with lovastatin; Table S2. Primers used for mutant strains; Table S3. Primers used for RT-qPCR.
