# Supplementary material for: The Role of Enoyl Reductase in the Monacolin K Biosynthesis Pathway in Monascus spp
Source: J Fungi (Basel). 2025 Mar 4;11(3):199. doi: 10.3390/jof11030199 (PMC11943018; doi:10.3390/jof11030199)
Supplement: Supplementary file 1 [file jof-11-00199-s001.zip › jof-3488439-supplementary.pdf]

Supplementary Materials

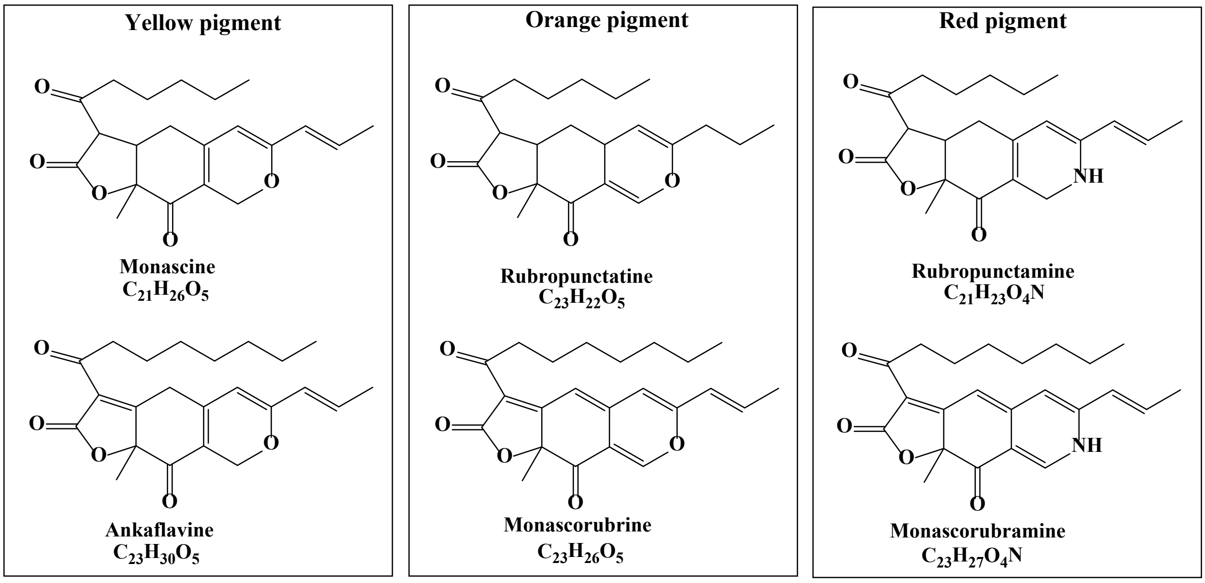

Figure S1. The structure of six typical MPs components

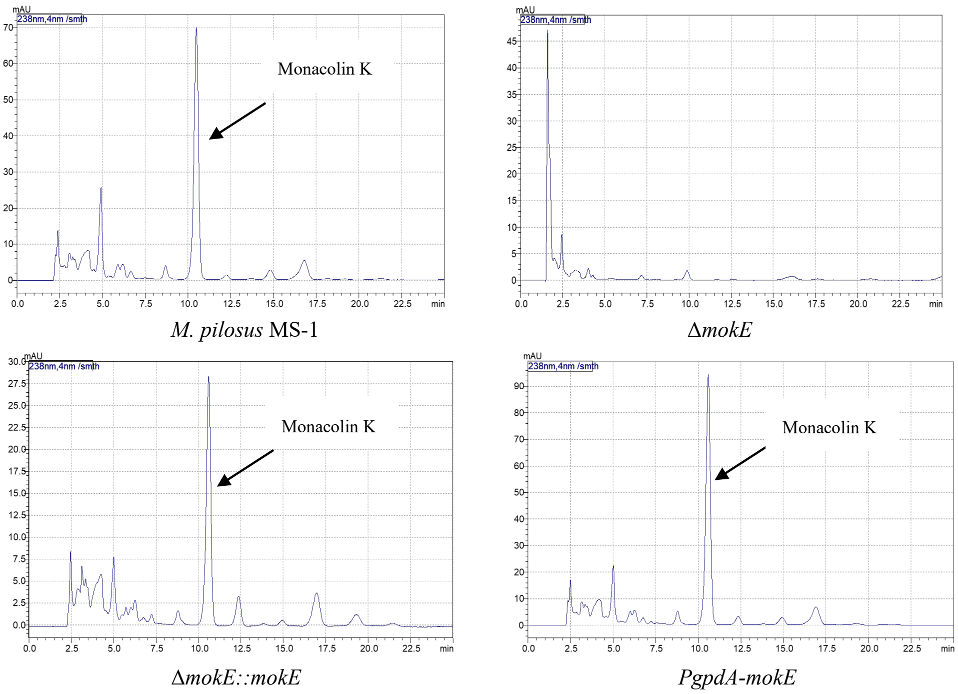

Figure S2. HPLC chromatograms of *M. pilosus* MS-1, Δ*mokE*, Δ*mokE::mokE* and *PgpdA-mokE* in MK on day 7.

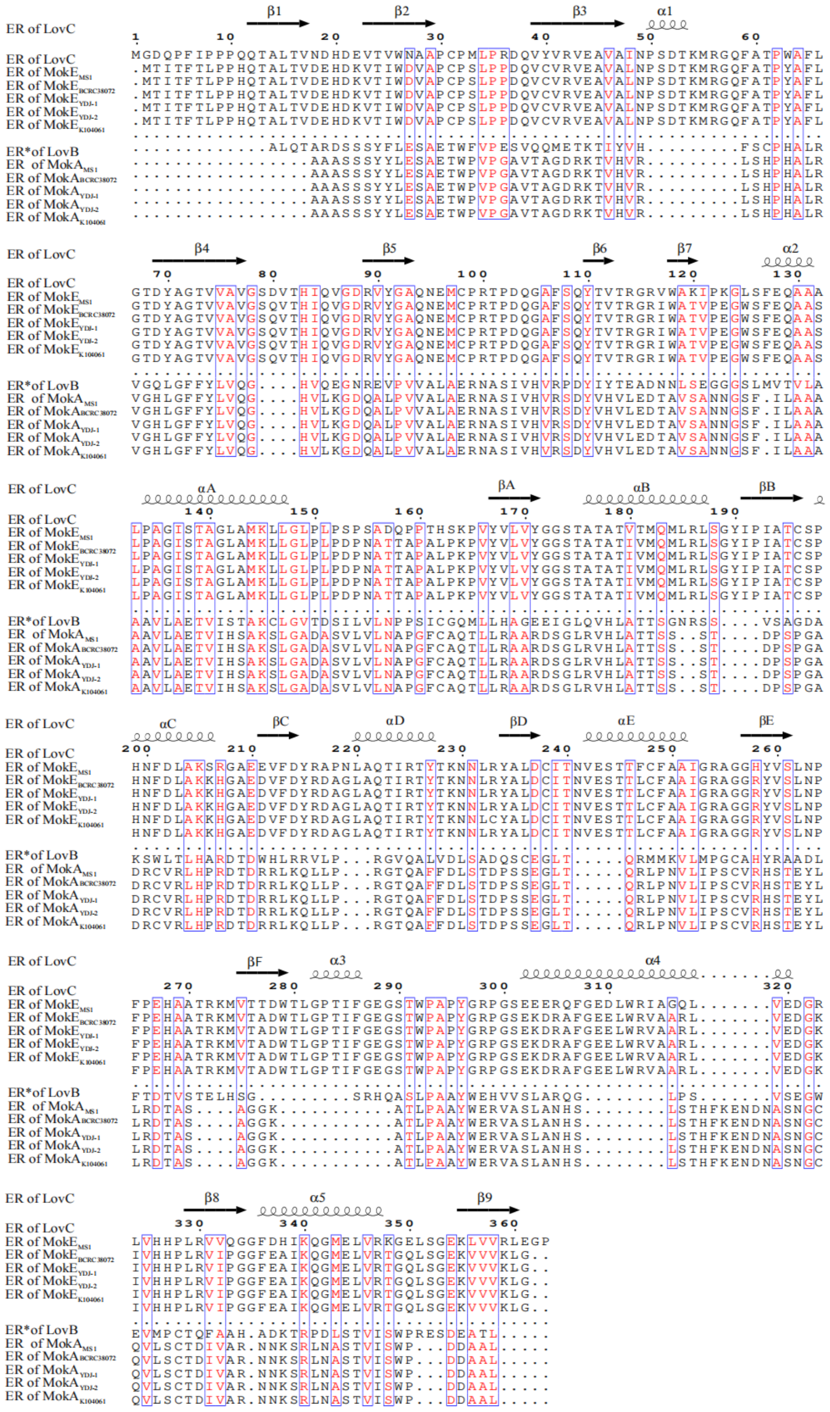

Figure S3. Alignments of ER domians of MokAs and LovB to those of MokEs and LovC

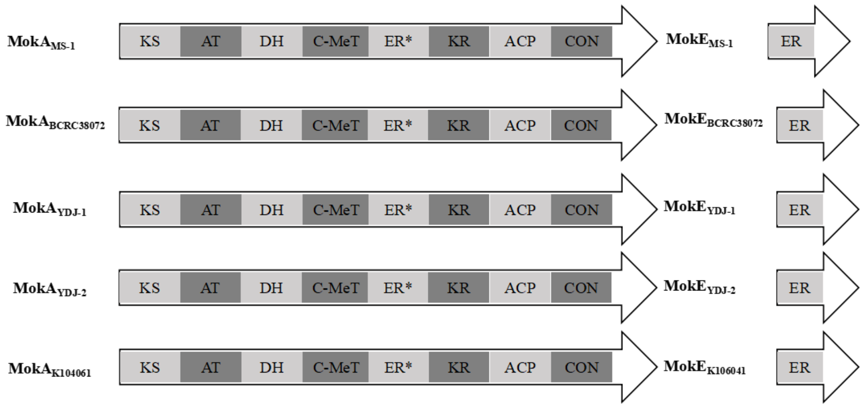

Figure S4. Domains of MokAs and MokEs from M. pilosus BCRC38072, MS-1, YDJ-1, YDJ-2 and K104061. KS: Ketoacyl synthase; AT: Acyltransferase; DH: Dehydratase; C-MeT: Carbon methyl transferase; ER: Enoyl reductase (ER* means inactive); KR: Ketoreductase; ACP: Acyl carrier protein; CON: Condensation.

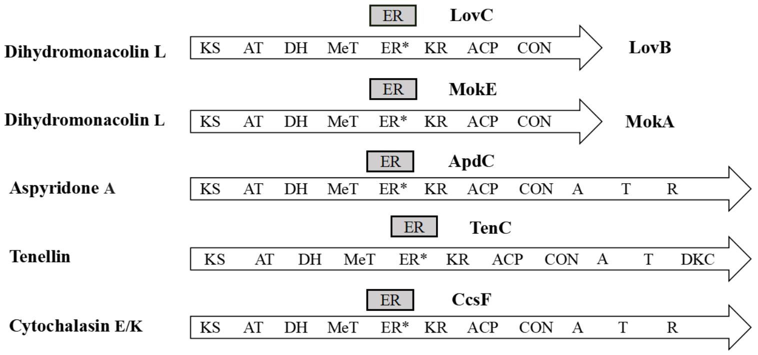

Figure S5. ER in closely related fungal PKS. ER*: an inactive state; KS: Ketoacylsynthase; AT: Acyltransferase; DH: Dehydratase, MT: Methyl transferase, ER: Enoyl reductase, KR: Ketoreductase, ACP: Acyl carrier protein, CON: Condensation, A: Adenylation, T: Thiolation, DKC: Dieckmann cyclase, R: reductase.

Table S1. Functional prediction of MK biosynthesis gene and homology comparison with lovastatin

***mok* genes**	**Functions**	**Homologous *lov* genes**	**Protein identities（%）**
mokA	Polyketide synthase	*lovB*	79
mokB	Polyketide synthase	*lovF*	72
mokC	P450 monooxygenase	*lovA*	86
mokD	Oxidoreductase	*lovG*	66
mokE	Enoyl reductase	*lovC*	84
mokF	Transesterase	*lovD*	88
mokG	HMG-CoA reductase	*lvrA*	72
mokH	Transcription	*lovE*	48
mokI	Efflux pump	*lovI*	81

Table S2. Primers used for mutant strains

Primers	Sequences（5′→3′）	Functions
mokE-5F	GGTACCCGGGGATCCTCTAGA<br>TCATAGTTCCTACAACTACACTACT	For amplification of 814 bp of 5′ flanking regions of *mokE*
mokE-5R	TCCTTCAATATCATCTTCTGTCGAC<br>GATGGGGATTACTATATGTGCTTAC	
hph-F	GTCGACAGAAGATGATATTG	For amplification of the *hph* gene (2137 bp)
hph-R	CTAGAAAGAAGGATTACCTC	
mokE-3F	GTTTAGAGGTAATCCTTCTTTCTAG<br>GATGCGTGTGTATGTATGTGTATGT	For amplification of 913 bp of 3′ flanking regions of *mokE*
mokE-3R	GGTACCCGGGGATCCTCTAGA<br>TCATAGTTCCTACAACTACACTACT	
mokE-ov5F	AAACCCGCATTATTCTGTCTTCTTC	PCR validation of deletion strains
mokE-ov5R	GCAGCTTCATACGAACTCTTCATCA	
HmokE-5F	GGTACCCGGGGATCCTCTAGA<br>AGAATACATGGCCAAAGGCC	For amplification of 2282 bp of 5′ flanking regions of *mokE* (containing *mokE* open reading frame region)
HmokE-5R	AGGCACTCTTTGCTGCTTGG<br>TACGCTCATTAGACCTTAACACAGG	
TtrpC-F	CCAAGCAGCAAAGAGTGCCTTCTAG	For amplification of the *TtrpC* gene (597 bp)
TtrpC-R	TTCGATGGGGTTGAGTTGG<br>CTAGAAAGAAGGATTACCTC	
G418-F	CCAACTCAACCCCATCGAACCGTAACC	For amplification of the neo gene (1221 bp)
G418-R	ATCATCATGCAACATGCATG	
HmokE-3F	CATGCATGTTGCATGATGAT<br>GATGCGTGTGTATGTATGTGTATGT	For amplification of 913 bp of 3′ flanking regions of *mokE*
HmokE-3R	ACGGCCAGTGCCAAGCTTGC<br>CCAGGGTATCCTACCCATCT	
HmokE-ov5F	GCATTATTCTGTCTTCTTCTCTTTC	PCR validation of complementation strains
HmokE-ov5R	AGTCTCCAACACAGTTGTTGAGTTT	
GmokE-5F	GGTACCCGGGGATCCTCTAGA<br>TGAGAAATTCGGGATCCAGAGTCGG	For amplification of 910 bp of 5′ flanking regions of *mokE*
GmokE-5R	TTCGATGGGGTTGAGTTGG<br>AAGACAGGATGGGTTCATGTGATTC	
gpdA-F	CATGCATGTTGCATGATGAT<br>GCCCCGAAGTGGAAAGGCTGGT	For amplification of the *gpdA* gene (604 bp)
gpdA-R	AAATAAAGGTTCTTGGATGGGAAGA	
GmokE-3F	CCATCCAAGAACCTTTATTT<br>TAAGCACATATAGTAATCCCCATCT	For amplification of 1779 bp of 3′ flanking regions of *mokE* (containing *mokE* open reading frame region)
GmokE-3R	ACGGCCAGTGCCAAGCTTGC<br>AGAAAGAGATCCGGAGAATCAGACT	
GmokE-ov5F	GCATTATTCTGTCTTCTTCTCTTTC	PCR validation of overexpression strains
GmokE-ov5R	TTGCGTAGACGAATTCAAAGTCTTC	

Labeled with single underline letters are partial sequences of vector pCAMBIA3300; Labeled with dotted lines letters are the partial sequences of the *hph* resistance gene; Labeled with wavy line letters are the partial sequences

of the G418 resistance gene; Labeled with double underline letters are the partial sequences of the *TtrpC* gene; Labeled with double wave underline letters are the partial sequences of the *gpdA* promoter.

Table S3. Primers used for RT-qPCR

Gene clusters	Gene	Gene function	Upstream primers（5′→3′）	Downstream primers（5′→3′）
	Beta-actin	Internal reference genes	CTGGCACCACACATTCTACAA	CGAAGACGATCTGGGTCATCT
MK	*mokA*	PKS	ACACGATCGGCATGTTCATG	CACCTGGATGACTTGGACCT
	mokB	PKS	GACATCGTACGTGGAAGCAC	CAGAGCCACCTTCATTACGC
	mokC	P450 monooxygenase	AACCGCTCCGTTTCATTGTC	CCTTGGTGATGAGATGTGCG
	mokD	Oxidoreductase	TCAGCACCAGGTCCAGAAAT	GGGCCATAAGTGTAGCATGC
	mokE	Dehydrogenase	GCCCGTGTATGTTTTGGTGT	CCGGACCTACACGAGATTGA
	mokF	Transeterase	TCGAGATCATAGTGGCCGAC	AGTCGATCCACCGTCTCATC
	mokG	HMG-CoA reductase	TGGGAGGCTTCATGACCAAT	TCCAACCCAGATGCTTCCAT
	mokH	Transcription factor	TGTGAGTATGGGCAAGGAGG	ATCAGCAGACTTGGCCTTCT
	mokI	Efflux pump	CTCGATCTCCTGGGGTTTGT	CAGAACCCAAACACCACCAG
MPs	*pigA*	PKS	CTCGAGGAATTGAGCGTTGG	CAGGAAGACTCAATTCGCCG
	pigB	Transcription factor	CAGAAACCATCACGCAGGAG	AAAGAAGCAGCGGGTCTACT
	pigC	Dehydrogenase	CCTACCCAGCAATCGATCCT	ACGTCCTTTGCTAGCTCTGT
	pigD	3-O-acetyltransferase	GTACGCGGGGAAGTTCAATC	CCCCAATATCCTCCCTCGTC
	pigE	Aryl-alcoholdehydrogenase	CTGTACAACGTCCTGCATCG	TCTCCCGAATCGTATCCAGC
	pigF	Amine oxidase	GTCACGTCTCAGATCGCAAC	CTGGCACTGTCGATGAACTG
	pigG	Oxydoreductase	TACAAGGAGTTCGGGCCATT	GCAGGCTAGCACACATCTTC
	pigH	Dehydrogenase	TCGTCTCGTGGATCATCTCG	GATGCTCTCCAATCCCTTGC
	pigI	Transcription factor	CATCTTGGACGGGATTGCAG	ATCTCGTCCTTGCTCACACA
	pigJ	Fatty acid synthase	CGTTTCGGCTGATCATTCGT	CGATCCCGCTGAAGAACTTG
	pigK	Fatty acid synthase	CAATCGGACGGGAAATGACC	CTTTGAGTCTCATCGCCAGC
	pigL	Ankyrin repeat protein	TCAGGGATTGTGGGATTGCT	CTTGCATCGCCTTGTCAACT
	pigM	P450 monooxygenase	GTGACTTTGAACAGCCTGGG	CGCTCAATTCCTTCTCCAGC
	pigN	Salicylate hydroxylase	CGATGCAATGGGGAGAGAGA	CGAATCCAGAGAAGGCTTGC
	pigO	Hypothetical protein	AACTGCTCTTCGAGACGGAT	CGAACTCCAGCAGCAACTTC
	pigP	Multidrug transporter	CTATTTGGTGCGGACGAGTG	TCCAACACCTCTTCGATGCT
